# Right Hemisphere Dominance for Unconscious Emotionally Salient Stimuli

**DOI:** 10.3390/brainsci11070823

**Published:** 2021-06-22

**Authors:** Elisabetta Làdavas, Caterina Bertini

**Affiliations:** 1 Centre for Studies and Research in Cognitive Neuroscience, University of Bologna, Via Rasi e Spinelli 176, 47023 Cesena, Italy; caterina.bertini@unibo.it; 2 Department of Psychology, University of Bologna, Viale Berti Pichat 5, 40121 Bologna, Italy

**Keywords:** fear, hemianopia, amygdala, implicit visual processing

## Abstract

The present review will focus on evidence demonstrating the prioritization in visual processing of fear-related signals in the absence of awareness. Evidence in hemianopic patients without any form of blindsight or affective blindsight in classical terms will be presented, demonstrating that fearful faces, via a subcortical colliculo-pulvinar-amygdala pathway, have a privileged unconscious visual processing and facilitate responses towards visual stimuli in the intact visual field. Interestingly, this fear-specific implicit visual processing in hemianopics has only been observed after lesions to the visual cortices in the left hemisphere, while no effect was found in patients with damage to the right hemisphere. This suggests that the subcortical route for emotional processing in the right hemisphere might provide a pivotal contribution to the implicit processing of fear, in line with evidence showing enhanced right amygdala activity and increased connectivity in the right colliculo-pulvinar-amygdala pathway for unconscious fear-conditioned stimuli and subliminal fearful faces. These findings will be discussed within a theoretical framework that considers the amygdala as an integral component of a constant and continuous vigilance system, which is preferentially invoked with stimuli signaling ambiguous environmental situations of biological relevance, such as fearful faces.

## 1. Introduction

Emotional facial expressions are particularly salient stimuli in our environment [1,2]. Faces provide various kinds of information about others, including identity and several emotional and motivational aspects. For instance, they may communicate the intentions of the other or the presence of a threat in the surroundings. Due to their intrinsic emotional and motivational value, emotional facial expressions are of particular interest. Among the different emotional expressions, fearful faces represent a special case of salient stimuli. Together with angry faces, fearful faces are negatively valenced and normally judged as very arousing. Both emotional expressions demand immediate attention from the observer to prepare respectively a fight or flight reaction [2]. At variance with angry faces, fearful faces are not intrinsically threatening, but they signal a potential upcoming danger in the environment, without specifying its nature or location [3,4]. In fact, fearful faces may act as a cue that prompts heightened perceptual sensitivity to threat in the environment [5]. In line with this hypothesis, fearful faces were found to enhance basic perceptual processes, such as contrast and orientation sensitivity and spatial resolution [6,7,8,9]. Since such a rapid detection of and response to threat is crucial for survival and has great adaptive value, it has been proposed that the mechanisms involved in threat detection and defense responses could depend on the existence of a specific neural fear system [10,11,12].

An important aspect of threat-related stimuli is their ability to elicit behavioral and neurophysiological responses without access to perceptual awareness. For instance, the processing of salient emotional stimuli, like threat-related stimuli—even when occurring outside the focus of attention—is given privileged and rapid access to attentional resources, compared to the processing of non-emotional stimuli (for a review, [2]), as demonstrated by facilitation in response times [1], or increased accuracy in identifying targets [13]. Indeed, the processing of threatening stimuli appears to be independent of goal-directed, top-down mechanisms, and seems to rely on automatic processes [14]. This is suggested by a large body of evidence showing, for instance, greater electrophysiological responses to salient emotional stimuli in indirect tasks (i.e., when participants are not engaged in the emotional component of the task or when the emotional content is irrelevant to the task; [15,16].

This review will present evidence showing that threat-related stimuli are preferentially processed, even in the absence of awareness, and are able to facilitate responses to visual stimuli in the surroundings. More precisely, evidence emerging from patients with lesions to the visual cortices and visual field defects will be reviewed, since they offer a unique model to explore the mechanisms and the neural underpinnings of unconscious threat implicit processing. In addition, this review will document the relevance of the right hemisphere for the visual processing of fearful facial expressions in the absence of awareness.

## 2. Processing of Fearful Signals in the Absence of Awareness in Patients with Visual Field Defects

Studies of patients with cerebral lesions, especially to the visual cortices, have provided important information about the properties and the neural underpinnings of non-conscious perception of emotional stimuli and the notion that processing of fearful stimuli is prioritized, compared to other emotional signals.

In a recent series of studies on implicit processing of emotional stimuli, patients with visual field deficits due to post-chiasmatic lesions typically performed at chance when they had to discriminate the visual features and the emotional content of stimuli presented in their blind field, or to guess the presence or absence of visual stimuli presented in the blind field [17,18,19,20]. However, when asked to perform a discrimination task of the emotional content of faces presented in their intact field while fearful faces were presented concurrently in their blind field, they nevertheless showed a response facilitation to consciously perceived faces in the intact field. In contrast, when happy or neutral faces were presented in the blind field, they failed to show a facilitatory effect. These results suggest that, in these patients, only fearful faces, but not happy or neutral faces, can be processed in the absence of awareness and can modulate responses towards consciously perceived stimuli in the intact field [18]. Similarly, fearful faces, but not happy faces, presented in the blind field increased the amplitude of the electrophysiological N170 component evoked by faces presented in the intact field, in line with evidence showing N170 modulations with highly salient [21,22], or unexpected [23,24] visual stimuli. It is known that the N170 reflects the early stages of structural encoding; thus, the increase in this electrophysiological component suggests that implicit fear processing might enhance the early visual encoding of faces presented in the intact field [20]. Moreover, the facilitatory effects of unseen fearful faces can also generalize to other visual stimuli outside the facial domain, as demonstrated by facilitatory effects in the discrimination of simple visual stimuli constituted by horizontal vs. vertical Gabor patches presented in the intact field [19]. Overall, these findings suggest that, in patients with lesions to the cortical visual pathway and visual field defects, fear-related visual information can be selectively processed in the absence of awareness and influence visual performance in the intact visual field.

Notably, the ability of hemianopic patients to selectively process fear in the absence of awareness appears to represent a different phenomenon from the abilities demonstrated by patients with affective blindsight [25,26,27]. Indeed, a limited number of patients with lesions to the primary visual cortex revealed the ability to discriminate between different emotional signals in the blind field (i.e., affective blindsight). More precisely, when they were asked to make a forced choice discrimination, contrary to the hemianopic patients described above, they were able to reliably discriminate the emotional content of fearful vs. happy, fearful vs. angry and angry vs. sad facial expressions presented in their blind field [25,27]. Moreover, they showed facilitatory effects in response to emotional stimuli in the intact field when concurrent and congruent emotional stimuli were presented in the blind field [26]. This pattern of performance—showing the ability to implicitly process a variety of emotional signals in different experimental tasks (both directly and indirectly tested in the blind field)—is distinct from the implicit processing demonstrated by hemianopics without blindsight (which is observed in indirect tasks and is fear-specific), and suggests that the abilities shown by patients with affective blindsight might be subserved by a peculiar plastic reorganization of both subcortical and cortical structures [28,29].

## 3. Implicit Fear-Related Processing in Hemianopics Is Mediated by a Subcortical Defensive Circuit

The behavioral and neurophysiological effects of the implicit processing of unseen fearful faces found in hemianopic patients without affective blindsight indicate that, when the visual cortex has been damaged or visual information cannot reach the visual cortex due to deafferentation, the only visual information available at the implicit level is related to fear. This implicit processing of fear information probably acts as a warning signal to enable rapid defensive responses.

This finding seems in line with the hypothesized existence of pre-wired defensive survival circuits capable of responding to species-specific threats [10]. These circuits are responsible for the emergence of multidimensional responses, such as behavioral and autonomic reactions and changes in brain activity, which might constitute a global defensive state [10].

An undoubtedly integral component participating in these threat-detection systems is the amygdala, as suggested by its critical role in responding to fear [30,31,32,33]. An influential model on emotional processing has postulated the existence of multiple pathways, involving both cortical and subcortical structures [34], that convey threat-related information to this subcortical structure. More precisely, the fine-grained and conscious evaluation of potentially dangerous stimuli has been proposed to rely on a cortical “high road” pathway, in which relevant visual information, originating from lower order visual regions, such as the thalamic lateral geniculate nucleus and the striate cortex, traverse the ventral temporal cortices to reach the amygdala [35] and, at a later stage, the prefrontal cortex for higher-level emotional and cognitive evaluation [36,37]. On the contrary, both human and non-human animals have evolved a direct pathway to the amygdala, bypassing the primary sensory cortices, which allows rapid and automatic responses to danger. Accordingly, a subcortical ‘low road’, encompassing the superior colliculus and the thalamic pulvinar and reaching the amygdala, has been proposed to mediate a fast but coarse visual analysis of potential threats [34]. Although some have argued against the existence of this subcortical route and have challenged the hypothesis of a rapid visual processing occurring in the subcortical structures involved in this pathway [38], recent findings have corroborated this view. Indeed, tractography studies, both in monkeys [39] and humans [28,39,40], have documented the existence of direct connections between the structures participating in this pathway. Moreover, electrophysiological findings have provided evidence of the involvement of these structures in rapid and unconscious visual processing of emotional stimuli [41,42,43]. Notably, fine-grained temporal resolution imaging techniques have confirmed very early responses to fearful faces in the amygdala, supporting the hypothesis of a quick and coarse transfer of visual information to this subcortical structure. Indeed, intracranial event-related potentials have demonstrated amygdala responses to fearful faces beginning 74 ms after stimulus onset [44], and magnetoencephalography has revealed gamma event-related synchronization in the amygdala as early as 20–30 ms after the presentation of faces expressing fear [45]. Consistent with this evidence and considering that this colliculo-pulvinar-amygdala circuit is usually spared after lesions to the primary visual pathway [46], it has been proposed that the implicit processing of fearful stimuli described in hemianopic patients might be subserved by the activity of this subcortical circuit. In line with this proposal, recent evidence has demonstrated that no facilitatory effects due to the implicit processing of fearful stimuli are observed if this subcortical pathway has been damaged by the lesion, as in hemianopic patients with additional lesions to the pulvinar [17]. In addition, evidence from healthy participants whose conscious perception of the emotional content of faces was prevented using backward masking also supported this hypothesis [47]. Indeed, fear-specific facilitatory effects resembling those found in hemianopics were observed after suppression of activity in the visual cortices by transcranial direct current stimulation (tDCS; [47]), therefore strengthening the evidence that the subcortical circuit is involved in implicit processing of threat-related visual information [48,49,50,51,52,53], in the absence of awareness and activity in cortical visual areas.

Importantly, the facilitatory effects observed in the presence of unaware fearful faces suggest that, when unconscious threat-related visual information reaches the amygdala, the consequent amygdala activation is capable of modulating connected sensory cortices, influencing sensory processing and, therefore, altering the gating of information processing. In other words, the activity of the amygdala in response to unaware threatening signals in the environment might be able to lower the threshold for detecting stimuli in the interconnected sensory cortices, facilitating the sensory analysis of the surroundings [54]. In this perspective, the amygdala, by enabling preferential processing of potentially threatening stimuli, can assure that information relevant to the organism influences behavior [55], so that adequate and adaptive responses are implemented [9].

## 4. Amygdala as the Key Component of a Vigilance System

In addition to evidence of responsiveness of the amygdala to a wide range of emotions [56,57,58] and to the valence emotional dimension [59,60], there is a large amount of evidence supporting the notion that the amygdala is the key player in fear responses. However, the exact role played by this structure in the complex chain of events that produces an emotional response remains an open question. The amygdala is believed to play a major role in orienting attention towards threat-related stimuli [2,61,62], integrating not only emotional but also spatial information. In line, this subcortical structure is considered an important component of a constant and continuous vigilance system, preferentially activated by arousing, ambiguous and biologically relevant sensory stimuli, in order to prepare adaptive responses to salient environmental changes [63,64,65,66]. For instance, greater activation of this subcortical structure was found in the presence of an unpredictable series of auditory tones, compared to a predictable series [67]. In this perspective, the amygdala acts to constantly monitor the environment for specific signals to modulate the vigilance level of the organism [54,68]. Accordingly, cortical EEG activation, a measure of an organism’s overall level of vigilance, has been shown to correlate with the neural activation of the amygdala in response to fear-related stimuli [69,70]. Moreover, in animals, electrical stimulation of the amygdala has been shown to produce an increase in the excitability of cortical neurons [71]. In line, the amygdala is considered part of an automatic alerting system [72], that involves also afferent and efferent projections to brainstem structures (e.g., locus coeruleus; [73,74]), directing noradrenergic stimulation in response to salient sensory input and facilitating the physiological mechanisms mediating rapid reactions to potential danger [74,75]. Importantly, among other biologically relevant stimuli, emotional faces expressing fear have been shown to induce preferential activation of the amygdala [33,58,76,77,78,79], therefore suggesting a prominent role for this class of stimuli in promoting personal safety and defensive responses. In line with this idea, the increased activity found in the amygdala in response to fearful faces seems not to be due to the general presence of a negative stimulus signaling threat, since angry faces (which are similarly negative and salient) elicit a weaker [77,80,81] and later [45] activation in this subcortical structure. Indeed, fearful and angry faces, despite having a similarly negative valence, convey different information about the location of the threat. More precisely, fearful facial expressions, unlike angry faces, do not represent a direct threat, but signal a potential upcoming danger in the environment, without specifying its nature or location [82]. In this respect, fearful facial expressions, in the absence of any other information about the location of the potential threat (e.g., eye gaze directed towards the threat), have been proposed to influence the distribution of spatial attention, eliciting an early fleeting attentional capture [83], followed by a later scanning of the surrounding environment to detect the source of the threat [83,84]. It was demonstrated, indeed, that fearful expressions strengthen the representation of contextual threat, eliciting vigilance in the visual periphery [85]. In line with these findings, a recent study by Ellena et al., [86,87] showed that, as fearful faces come closer to the body, at variance with neutral and happy faces, they promote a redirection of attention towards the periphery. Instead, when angry faces, which represent a threat per se, come closer to the subject, attention is directed towards the angry face, leaving any peripheral event unattended (Ellena et al., in preparation).

This converging evidence therefore strengthens the notion that the amygdala is a crucial component in a system responding to ambiguous and relevant environmental stimuli (such as fearful faces). Interestingly, the aforementioned fear-specific, implicit processing observed in hemianopics seems to provide an indirect confirmation of this view. Indeed, the facilitatory effect of unseen fearful faces in hemianopics was evident only when the non-conscious, fear-related stimulus was presented in an ambiguous experimental condition (i.e., when emotionally incongruent happy or neutral faces were concurrently presented as a target in the intact field). In contrast, no effect was observed when a congruent fearful face was displayed in the intact field [18,20]. In the same vein, hemianopic patients also showed a reduction in reaction times to simple visual stimuli (Gabor patches) seen in their intact field during the concurrent presentation of unseen fearful faces in the blind field, again suggesting that non-conscious, threat-related information facilitates visual performance when ambiguous, non-redundant information is presented in the intact field [19].

As a whole, these findings corroborate the existence of a continuous monitoring system, in which the amygdala can boost vigilance in response to uncertain events which might potentially signal the presence of a threat, in order to allocate spatial attention and modulate visual analysis and exploration [88].

## 5. The Contribution of the Right Hemisphere to Implicit Fear Processing

An intriguing finding concerning the previously mentioned implicit processing of fearful faces in patients with hemianopia was that the facilitatory effects of presenting fearful faces in the blind field were found only after lesions to the visual cortices in the left hemisphere. Indeed, both the behavioral facilitation in discriminating visual stimuli in the intact field [17,19] and the enhancement of the N170 component reflecting the visual encoding of faces in the intact field [20], while fearful faces were concurrently presented in the blind field, were evident only in left-lesioned hemianopics. In contrast, hemianopic patients with damage to the right hemisphere did not show any sign of fear-related processing in the absence of awareness [17,19,20]. This finding suggests that the subcortical pathway in the right hemisphere conveying threat-related, visual information from the superior colliculus to the amygdala via the pulvinar [28,39,40] might be more relevant to the implicit processing of fear-related stimuli than the same pathway in the left hemisphere.

In line with this hypothesis, neuroimaging findings showed that significant neural responses were elicited in the right but not the left amygdala by unconscious fear-conditioned stimuli [89]. Similarly, lateralized activations of right visual areas and the right amygdala have been found after presentation of unattended fearful faces in the left visual field, while presentation in the right visual field did not reveal similar lateralized activations [90]. Moreover, unattended fearful facial expressions have been shown to induce early activations in the right amygdala (as fast as 100 ms after stimulus presentation), while activation in the left amygdala occurred at later stages, supporting the role of the right amygdala in rapidly responding to implicit threats [91]. This also seems consistent with the hypothesis that the right amygdala is involved in fast and reflexive responses to threat, as suggested by the finding that right amygdala responses to fearful faces occur earlier but are more prone to habituation (i.e., rapid decreases in amplitude) compared with left amygdala responses [92].

Moreover, connections between the right amygdala and the structures participating in the subcortical emotional pathway (i.e., the superior colliculus and the pulvinar) in the right hemisphere have been shown to play a key role in unconscious fear perception. Neuroimaging evidence showed that connectivity between the right amygdala, the pulvinar and the superior colliculus was increased by the presentation of masked fear-conditioned faces, while the connectivity between the left amygdala and the pulvinar, or the superior colliculus, did not discriminate between the presentation of seen or unseen fear-conditioned targets [93]. Moreover, conscious and unconscious fear perception have been shown to be subserved by different and asymmetric functional connectivity patterns [36]. Indeed, conscious fear perception was linked with negative functional connectivity between both the left and right amygdala, the thalamic lateral geniculate nucleus and the striate cortex, reflecting reentrant feedback connections related to visual awareness. In contrast, unconscious fear perception elicited positive functional connectivity between the right amygdala, the superior colliculus and the pulvinar, reflecting excitatory feedforward connections supporting rapid processing of threat and suggesting the active engagement of the right subcortical emotional pathway in processing fear in the absence of awareness [36]. More recently, connectivity within this right-lateralized subcortical pathway has also been demonstrated to be directly associated with orienting towards unattended threats [40]. Indeed, a group of healthy participants—in whom diffusion weighted magnetic resonance imaging (DTI) confirmed the existence of a direct subcortical pathway from the retina to the amygdala via the superior colliculus and the pulvinar [40]—was asked to perform a temporal order judgment saccade decision task, where participants were presented with threatening and non-threatening images in both visual fields and were asked to make a saccade toward the image that appeared first. Although the emotional content of the pictures was irrelevant to the task, participants showed a bias to orient towards threat, and fractional anisotropy (FA) measurements revealed that this bias was predicted by individual differences in the microstructure of connections in the colliculo-pulvinar-amygdala pathway in the right hemisphere, but not the left [40]. In line with this observation, fiber density and structural connectivity between the pulvinar and the amygdala in the right hemisphere are also related to recognition of fearful expressions [94].

## 6. Concluding Remarks

The evidence reviewed in the previous paragraphs used patients with visual field defects and unilateral lesions to the primary visual cortices as a model to investigate implicit visual processing of emotional stimuli in the absence of awareness and the contribution of subcortical visual circuits to this phenomenon. The reported findings highlight that, after lesions to the primary visual pathway, fearful signals receive preferential implicit processing that can facilitate responses to visual stimuli presented in the intact visual field. This suggests the presence of an adaptive mechanism through which evolutionarily relevant visual information can be processed without awareness, promoting effective visual exploration of the environment to engage defensive responses to possible threats. Moreover, the colliculo-pulvinar-amygdala pathway in the right hemisphere seems to account for such fear-specific, implicit processing, since only hemianopics with lesions to the left-hemisphere (i.e., with a sparing of the right hemisphere) show these effects.

The dominance of the right hemisphere in processing and responding to emotional stimuli has been theorized (i.e., the right hemisphere hypothesis; [95,96]) based on several classical experiments and clinical data. More recently, this view has been updated, proposing a specialization of the right hemisphere in the processing of emotional stimuli occurring outside the focus of awareness, which has been widely documented and supported by a large amount of behavioral and neuroimaging studies of both healthy individuals and brain-damaged patients [97,98]. For instance, in neurologically intact participants, the influence of masked emotional expressions over conscious recognition of emotions has been shown to be stronger when the unseen emotional content was projected into the left visual field (and processed by the right hemisphere), compared to the right visual field [99]. Similar lateralized effects were also found with subliminal affective priming paradigms [100], and with morphed hybrid faces with subliminal emotional expressions [101,102]. In addition, studies of split-brain patients who underwent surgical resection of the corpus callosum have offered the unique opportunity to directly test cerebral asymmetries in implicit emotion processing and have revealed that non-conscious emotional stimuli, contrary to conscious emotional stimuli, can elicit appropriate autonomic responses [103] and influence explicit responses [104] only when briefly presented in the left visual field (i.e., processed by the right hemisphere), further supporting the higher sensitivity of the right hemisphere to non-conscious emotional processing.

However, the specific, implicit processing of unseen fearful stimuli observed only in hemianopics with left hemispheric lesions [17,18,19,20], and the greater responsiveness of the right subcortical pathway—from the superior colliculus to the amygdala via the pulvinar—to threat-related visual information [28,39,40,89], seem to suggest not only a role for the right hemisphere in unconscious emotional processing, but also a specialized preference for processing non-conscious, fear-related information.

Although further research is needed to corroborate and provide a conclusive interpretation of this right-hemisphere specialization for unconscious fear-related information, the pivotal contribution of the amygdala, which is an integral part of the subcortical emotional circuit, might help explain this fear-specific, implicit effect. Indeed, the amygdala is crucial for responding to ambiguous and uncertain salient environmental stimuli and plays a role in boosting vigilance, which might help lower activation thresholds throughout sensory cortices and deploy attention to the surroundings to search for potentially dangerous signals [54,88]. From this perspective, interconnected attentional networks [105,106], regulating attentional allocation in visual space, might participate in these defensive mechanisms, promoting effective scanning and visual analysis of the environment. Thus, the well-known dominance of the right hemisphere in visuo-spatial performance [107,108,109,110,111,112], and attentional allocation [105,106,113,114,115], might have favored connections and reciprocal signaling between the emotional subcortical circuit and the attentional networks within the same hemisphere, therefore strengthening the right-lateralized prevalence in rapid and unconscious detection of threat.
